# Supplementary material for: Pyruvate Dehydrogenase Kinase 1 inhibition mediated oxidative phosphorylation enhancement in cartilage promotes osteoarthritis progression
Source: BMC Musculoskelet Disord. 2023 Jul 20;24:597. doi: 10.1186/s12891-023-06585-6 (PMC10357736; doi:10.1186/s12891-023-06585-6)
Supplement: Supplementary file 1 — Additional file 1. [file 12891_2023_6585_MOESM1_ESM.zip › MF.Gsea.1653627316242/GOMF_TRANSFERASE_ACTIVITY_TRANSFERRING_NITROGENOUS_GROUPS.html]

Details for gene set GOMF\_TRANSFERASE\_ACTIVITY\_TRANSFERRING\_NITROGENOUS\_GROUPS[GSEA]

|  || Dataset | 55457\_collapsed\_to\_symbols.cls.cls #OA\_versus\_normal.cls.cls #OA\_versus\_normal\_repos |
| Phenotype | cls.cls#OA\_versus\_normal\_repos |
| Upregulated in class | OA |
| GeneSet | GOMF\_TRANSFERASE\_ACTIVITY\_TRANSFERRING\_NITROGENOUS\_GROUPS |
| Enrichment Score (ES) | 0.3718071 |
| Normalized Enrichment Score (NES) | 1.1668148 |
| Nominal p-value | 0.26086956 |
| FDR q-value | 0.61580986 |
| FWER p-Value | 1.0 |
Table: GSEA Results Summary

  

Fig 1: Enrichment plot: GOMF\_TRANSFERASE\_ACTIVITY\_TRANSFERRING\_NITROGENOUS\_GROUPS      
 Profile of the Running ES Score & Positions of GeneSet Members on the Rank Ordered List

  

| SYMBOL | TITLE | RANK IN GENE LIST | RANK METRIC SCORE | RUNNING ES | CORE ENRICHMENT || 1 | GOT1 | glutamic-oxaloacetic transaminase 1 [Source:HGNC Symbol;Acc:HGNC:4432] | 59 | 0.974 | 0.2371 | Yes |
| 2 | GOT2 | glutamic-oxaloacetic transaminase 2 [Source:HGNC Symbol;Acc:HGNC:4433] | 1308 | 0.457 | 0.2535 | Yes |
| 3 | TAT | tyrosine aminotransferase [Source:HGNC Symbol;Acc:HGNC:11573] | 1979 | 0.363 | 0.2915 | Yes |
| 4 | OAT | ornithine aminotransferase [Source:HGNC Symbol;Acc:HGNC:8091] | 2074 | 0.353 | 0.3718 | Yes |
| 5 | BCAT2 | branched chain amino acid transaminase 2 [Source:HGNC Symbol;Acc:HGNC:977] | 3552 | 0.198 | 0.3063 | No |
| 6 | KYAT3 | kynurenine aminotransferase 3 [Source:HGNC Symbol;Acc:HGNC:33238] | 4128 | 0.143 | 0.2972 | No |
| 7 | GFPT1 | glutamine--fructose-6-phosphate transaminase 1 [Source:HGNC Symbol;Acc:HGNC:4241] | 5847 | 0.005 | 0.1651 | No |
| 8 | GFPT2 | glutamine-fructose-6-phosphate transaminase 2 [Source:HGNC Symbol;Acc:HGNC:4242] | 5849 | 0.005 | 0.1663 | No |
| 9 | PSAT1 | phosphoserine aminotransferase 1 [Source:HGNC Symbol;Acc:HGNC:19129] | 6585 | -0.049 | 0.1213 | No |
| 10 | ABAT | 4-aminobutyrate aminotransferase [Source:HGNC Symbol;Acc:HGNC:23] | 6804 | -0.063 | 0.1201 | No |
| 11 | ETNPPL | ethanolamine-phosphate phospho-lyase [Source:HGNC Symbol;Acc:HGNC:14404] | 6996 | -0.079 | 0.1250 | No |
| 12 | BCAT1 | branched chain amino acid transaminase 1 [Source:HGNC Symbol;Acc:HGNC:976] | 7106 | -0.088 | 0.1384 | No |
| 13 | AGXT | alanine--glyoxylate and serine--pyruvate aminotransferase [Source:HGNC Symbol;Acc:HGNC:341] | 8115 | -0.164 | 0.1008 | No |
| 14 | GPT | glutamic--pyruvic transaminase [Source:HGNC Symbol;Acc:HGNC:4552] | 8411 | -0.186 | 0.1241 | No |
| 15 | KYAT1 | kynurenine aminotransferase 1 [Source:HGNC Symbol;Acc:HGNC:1564] | 8633 | -0.203 | 0.1573 | No |
| 16 | AMT | aminomethyltransferase [Source:HGNC Symbol;Acc:HGNC:473] | 9021 | -0.234 | 0.1852 | No |
| 17 | GAPDH | glyceraldehyde-3-phosphate dehydrogenase [Source:HGNC Symbol;Acc:HGNC:4141] | 11268 | -0.467 | 0.1267 | No |
Table: GSEA details [plain text format]

  

Fig 2: GOMF\_TRANSFERASE\_ACTIVITY\_TRANSFERRING\_NITROGENOUS\_GROUPS      
 Blue-Pink O' Gram in the Space of the Analyzed GeneSet

  

Fig 3: GOMF\_TRANSFERASE\_ACTIVITY\_TRANSFERRING\_NITROGENOUS\_GROUPS: Random ES distribution      
 Gene set null distribution of ES for **GOMF\_TRANSFERASE\_ACTIVITY\_TRANSFERRING\_NITROGENOUS\_GROUPS**
